# Supplementary material for: Assessing Versatile Machine Learning Models for Glioma Radiogenomic Studies across Hospitals
Source: Cancers (Basel). 2021 Jul 19;13(14):3611. doi: 10.3390/cancers13143611 (PMC8306149; doi:10.3390/cancers13143611)
Supplement: Supplementary file 1 [file cancers-13-03611-s001.zip › Supplementary_materials_210712_revision.pdf]

Assessing versatile machine learning models for glioma radiogenomic studies across hospitals

Risa Karakida Kawaguchi, Masamichi Takahashi, Mototaka Miyake, Manabu Kinoshita, Satoshi Takahashi, Koichi Ichimura, Ryuji Hamamoto, Yoshitaka Narita, and Jun Sese.

1 PABLO: publicly available brain image analysis toolkit

This study aims to establish a comparable benchmark for general glioma radiogenomic studies. The pipeline used in this study, which contains scripts for other applications, is also publicly available in our github repository (https://github.com/carushi/pablo). PABLO consists of four steps: (1) Brain embedding, (2) Pixel normalization, (3) Radiomic feature computation, and (4) Classification. We describe the details of each step in the following subsections. Due to the high identifiability of brain MRI data, certain modification and preprocessing steps, such as image-image alignment, resampling, and skull-stripping, have already been applied to TCIA data. On the other hand, the computational environment required to apply exactly the same modifications to the local dataset is not always available because radiogenomic pipelines tend to be made privately, in-house, and are complex due to the combination of many applications.

1.1 Brain embedding

PABLO contains scripts to apply brain embedding using SPM12 [1] and ANTs [2]. Such brain embedding steps have been shown to increase the accuracy of automated segmentation due to the reduction of systematic biases [3]. We also found that the systematic biases of radiomic features for each dataset are also mitigated by use of brain embedding (Figure 2 in the main text). For this reason, we compared prediction accuracy with and without brain embedding.

1.2 Pixel normalization

Due to the characteristics of relative MRI quantification, the pixel distribution of MRI and other advanced MRI may differ for each sequence [4]. To standardize pixel distributions, two methods of pixel normalization were applied. The first method,

outlier filtering, involves removing the highest 0.1% and negative pixels, then all remaining pixels were scaled into a range from 0 to 255. The highest and negative pixels were set to 255 and 0, respectively. The second method, z-score filtering, consists of three steps: (1) converting the pixels into z-scores using the average and standard deviation, (2) removing values with absolute Z-scores greater than 3, and (3) normalizing the remaining values into a range from 0 to 255 following the equation $((x - min) \times 255/(max - min))$, where min and max correspond to the minimum and maximum values of pixels for the whole brain. Each value excluded at the second step was set to 255 (0) when it was positive (negative). We also applied skull-stripping using FSL[5] before normalization to remove pixels that are too bright from bones. Specifically, standard_space_roi in the FSL library is used for brain images without brain embedding. For the other, embedded brains, the brain regions are selected according to the standard brain space.

1.3 Radiomic feature computation

In this study, we combined radiomic features, including texture-based features, and deep learning (DL)-based features computed for four types of MRI sequences with the patients' clinical information as described below.

1. Basic features (28×4 dimension): Statistics of pixel values from four types of MRI scans. Twenty-eight features are generated from each MRI type. These features consist of percentiles from 0 to 100 in 10% increments for the pixel values of the tumor (edema) and whole regions, as well as several statistics such as mean, median, min, max, dimension size (□, □, □), and centroid coordinates of the whole brain.
2. Pyradiomics-based features (960×4): first order statistics, shapes, and textures are calculated by pyradiomics software [6]. Pyradiomics, which is frequently used for radiomic analysis, was used for radiomic feature extraction from 2D and 3D images with the region of interest (ROI) information. All parameters were computed except for NGTDM due to the long running time.
3. Pre-trained deep learning (DL)-based features (3072×4). The DL model Inception-Resnet v2 [7], pre-trained by ImageNet database, was used to obtain general image features. The outputs of the second last layer were used . Because the

number of slices containing the tumor is different for each patient, averages and sums of the outputs along the z-axis were calculated. The input image consists of a portion of the MRI scan that is cropped only around the tumor or edema regions.

4. Anatomical tumor location (30). A vector representing occupancies of each anatomical region was used to indicate 3D tumor position using the “Atlasquery” command equipped in FSL. As a standard atlas, Harvard-Oxford Subcortical Structural Atlas and MNI Structural Atlas were applied.
5. Clinical information (3). Features such as sex, age, and KPS (Karnofsky Performance Status) for each patient were applied to represent patients’ clinical information. Female and male features are converted into 1 and 2, respectively. In this study, KPS is a score ranging from 0 to 100 with a step value of 10.

In cases where any image or clinical information could not be obtained, the vector is filled with zeros. To integrate clinical information with image features, we additionally normalized the vectors of each feature from patients within one dataset prior to classification. Image features are computed for whole brain regions if no ROI information is provided. In total, 16,221 features are computed and applied for prediction.

1.4 Classification

To generate a machine learning model for glioma MRI scans, we applied a variety of machine learning methods; logistic regression with lasso penalty (Logistic, □ = 0.1, 0.5, 1.0), linear discriminant analysis (LDA), k-nearest neighbor (kNN, □ = 1, 3, 5), random forest (RF), xgboost (XGB), Adaboost (AB), and support vector machine (SVM). Before classification, two different dimension reduction methods PCA and NMF were also applied to extract robust features from a number of radiomic features. The dimension of the input matrix after compression is set to 8, 20, 40, and 200. If dimension reduction is not applied (represented as Feature selection), only Logistic and LDA are utilized for the prediction because the training of other classifiers is not feasible for such a large input matrix.

2 Tools used in this study

1. SPM v12 [1]

2. ANTs: Advanced Normalization Tools v1.9 [2]
3. Pyradiomics v2.2.0 [6]
4. FSL v5.0.10 [5]

3 Abbreviation

- MRI: magnetic resonance imaging
- ROI: region of interest
- GBM: glioblastoma multiform
- LrGG: lower grade glioma
- LDA: linear discriminant analysis
- kNN: k-nearest neighbor
- RF: random forest
- XGB: XGBoost
- AB: AdaBoost
- SVM: support vector machine
- PCA: principle component analysis
- NMF: non-negative matrix factorization
- AUROC: area under receiver operating characteristic curve

4 Table and Figures

Supplementary Table S1. Patient demographics in each cohort. The definition of Group A, B, C, and D follow [8].

Supplementary Table S2. Summary of MRI scanners and scan protocols for each MRI data.

Supplementary Table S3. Summary of p-values for the comparison of ROC curves used in the main figures. The p-values are computed for each pair of ROC curves using roc.test function in pROC library with a "bootstrap" option. The ROC curves are approximated by the 50 points sampled from each real (average) ROC curve for the TCIA cross validation, NCC validation, and NCC test datasets. The number of positive and negative sample size is set to the half of positive and negative samples in the NCC validation and test, and one fifth of them in the TCIA dataset, respectively.

Supplementary Figure S1. (A) AUROCs from our machine learning workflow that used tumor ROI information to obtain radiomic features without brain embedding. Predictions were applied to two scenarios; Existence of the IDH1/2 mutation and prediction of MGMT methylation status. TERT promoter methylation prediction and chr1p19q co-deletion prediction cannot be carried out as there is a lack of observations for cross validation purposes. The accuracy of the cross validation results in TCIA, as well as the application of the model to the NCC validation set (NCC valid) and the NCC test set (NCC test) are indicated by blue, orange and gray bars, respectively. (B) and (C) AUROC comparison for prediction of IDH mutation and MGMT methylation under the same conditions described in (A), with the exception that a different dimension reduction method PCA (B) and NMF (C) was applied, respectively.

Supplementary Figure S2. Classification performance comparison between the highest AUROC model in TCIA and NCC validation for the prediction of IDH mutation (A) and MGMT methylation (B) under the same conditions as Supplementary Figure 1. Charts on the left show AUROCs of the model whose AUROC is the highest under cross validation with the TCIA dataset, while charts on the right denote AUROCs of the model whose AUROC is highest within the NCC validation set.

Supplementary Figure S3. (A) AUROCs of our machine learning workflow that excluded both ROI information or brain embedding on five prediction scenarios; GBM/LrGG classification, existence of the IDH1/2 mutation, MGMT methylation status prediction, TERT promoter methylation prediction and chr1p19q co-deletion prediction. Accuracy of the cross validation results in TCIA, as well as on the application of the model to the NCC validation set (NCC valid) and the NCC test set (NCC test) are indicated by blue, orange and gray bars, respectively. (B) and (C) Performance for GBM classification (B) and IDH mutation prediction (C) when standardization methods are changed under the same conditions described in (A). Blue, orange and gray bars corresponds to the AUROCs for TCIA cross validation, NCC validation and NCC test accuracies, respectively.

Supplementary Figure S4. Performance for GBM classification and IDH mutation prediction when the dimension reduction methods are changed under the same conditions described in Supplementary Figure 3. AUROC changes in GBM/LrGG classification (A) and IDH1/2 mutation prediction (B) due to differences in dimension

reduction methods. Blue, orange and gray bars corresponds to TCIA cross validation, NCC validation, and NCC test accuracies, respectively.

Supplementary Figure S5. (A) and (B) Comparison of performance between the highest AUROC model in TCIA and NCC validation for GBM/LrGG classification (A) and IDH1/2 mutation prediction (B) under the same conditions described in Supplementary Figure 4. Charts on the left show AUROCs of the model whose AUROC is the highest under cross validation within the TCIA dataset. Charts on the right denote the AUROCs of the model whose AUROC is the highest within the NCC validation set. (C) AUROCs of our machine learning workflow without ROI information applied to five prediction scenarios; GBM/LrGG classification, IDH1/2 mutation existence, MGMT methylation status prediction, TERT promoter methylation prediction, and chr1p19q co-deletion prediction. The experimental conditions are the same as that described in Supplementary Figure 3-4, with the exception that the data contains clinical information. AUROC of the cross validation results in TCIA, as well as on the application of the model to the NCC validation set (NCC valid) and to the NCC test set (NCC test), are indicated by green, blue, and yellow bars, respectively.

Supplementary Figure S6. (A) and (B) Performance for GBM classification using each single feature for different feature computation methods (A) and basic features computed for the pixels within ROI (tumor) and entire region (all) (B). The AUROCs are computed for all NCC dataset. The AUROCs lower than 0.5 are converted to the values that are equal or larger than 0.5 by the equation 1.0-AUROC.

Supplementary Figure S7. (A) and (B) Performance for GBM classification and IDH mutation prediction by eight different machine learning classifiers for the whole NCC dataset. AUROCs of GBM/LrGG classification (A) and IDH1/2 mutation prediction (B) are computed for the NCC dataset without dividing the dataset into the test and validation set. Blue plots represent the AUROCs produced by classifiers trained on the TCIA dataset while orange plots represent AUROCs of cross validation for the NCC dataset. The prefix "F-" indicates results of feature selection obtained without applying the dimension reduction methods. (C) and (D) Classification performances of GBM prediction by eight different machine learning classifiers for the whole NCC dataset. AUROCs of GBM/LrGG classification for the whole NCC dataset are computed with or without standardization and dimension reduction methods. The x-axis corresponds to AUROCs from cross validation within the TCIA dataset and the y-axis represents

AUROCs produced from the NCC dataset using classifiers trained on the TCIA dataset (C) and AUROCs of cross validation within the NCC dataset (D).

References

[1] Friston KJ, Ashburner JT, Kiebel SJ, Nichols TE, and Penny WD. Statistical parametric mapping: The analysis of functional brain images. 2007.

[2] Brian B Avants, Nick Tustison, and Gang Song. Advanced normalization tools (ANTs). Insight j, 2:1–35, 2009.

[3] K Kazemi and N Noorizadeh. Quantitative comparison of SPM, FSL, and Brainsuite for brain MR image segmentation. Journal of biomedical physics & engineering, 4(1):13, 2014.

[4] Wieke Haakma, Jeroen Hendrikse, Lars Uhrenholt, Alexander Leemans, Lene Warner Thorup Boel, Michael Pedersen, and Martijn Froeling. Multicenter reproducibility study of diffusion MRI and fiber tractography of the lumbosacral nerves. Journal of Magnetic Resonance Imaging, 48(4):951–963, 2018.

[5] Mark Jenkinson Christian, F. Beckmann, Timothy E.J. Behrens, Mark W. Woolrich, and Stephen M.Smith. FSL. NeuroImage, 62(2):782–90, 2012.

[6] Joost JM van Griethuysen, Andriy Fedorov, Chintan Parmar, Ahmed Hosny, NicoleAucoin, Vivek Narayan, Regina GH Beets-Tan, Jean-Christophe Fillion-Robin, Steve Pieper, and Hugo JWL Aerts. Computational radiomics system to decode the radiographic phenotype. Cancer research, 77(21):e104–e107, 2017.

[7] Christian Szegedy, Sergey Ioffe, Vincent Vanhoucke, and Alexander A. Alemi. Inceptionv4, inception-resnet and the impact of residual connections on learning. AAAI 2017 proceedings, 4278–4284, 2017.

[8] Hideyuki Arita, Kai Yamasaki, Yuko Matsushita, Taishi Nakamura, Asanao Shimokawa, Hirokazu Takami, Shota Tanaka, Akitake Mukasa, Mitsuaki Shirahata, Saki Shimizu, et al. A combination of TERT promoter mutation and MGMT methylation status predicts clinically relevant subgroups of newly diagnosed glioblastomas. Acta neuropathologica communications, 4(1):79, 2016.
