# Supplementary figures and images for: Assessing Versatile Machine Learning Models for Glioma Radiogenomic Studies across Hospitals
Source: Cancers (Basel). 2021 Jul 19;13(14):3611. doi: 10.3390/cancers13143611 (PMC8306149; doi:10.3390/cancers13143611)

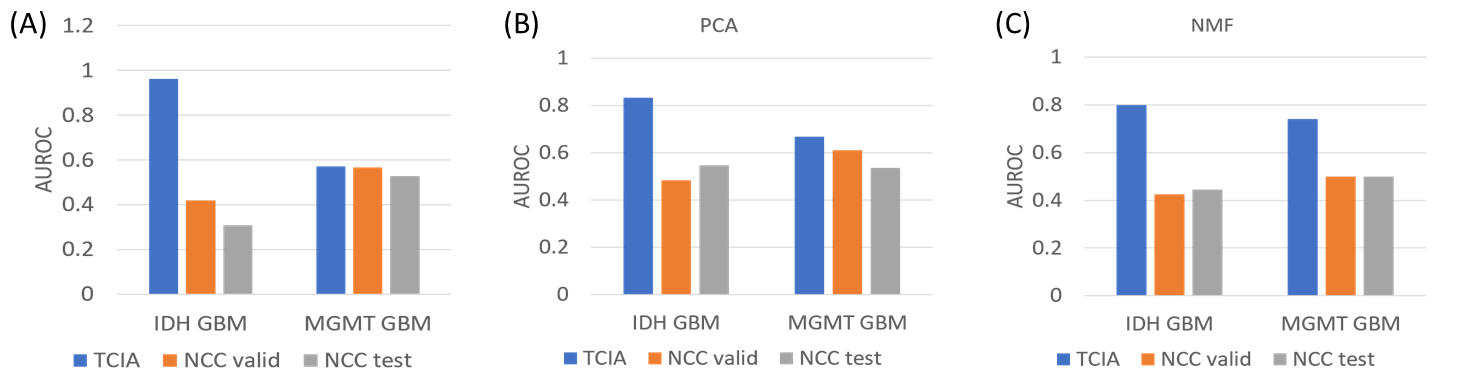

Supplement: Supplementary file 1 [file cancers-13-03611-s001.zip › Supplementary_Figure_S1.tif]

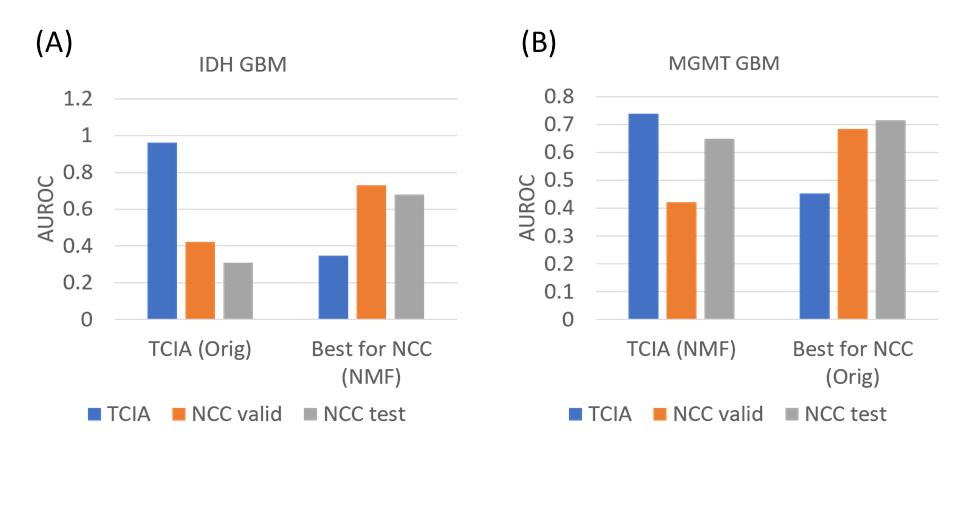

Supplement: Supplementary file 1 [file cancers-13-03611-s001.zip › Supplementary_Figure_S2.tif]

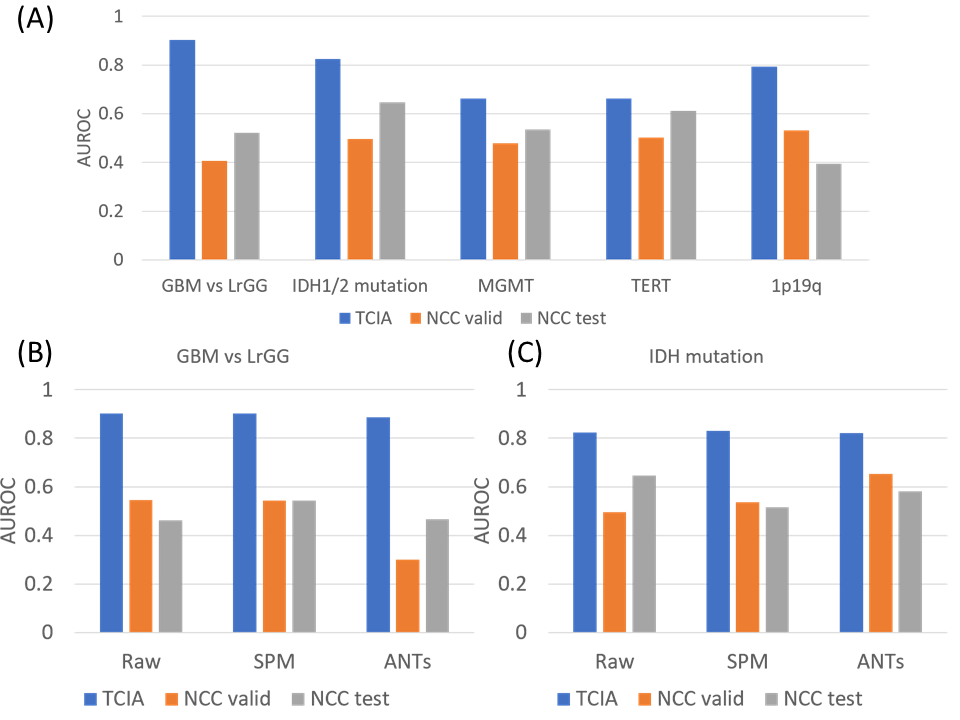

Supplement: Supplementary file 1 [file cancers-13-03611-s001.zip › Supplementary_Figure_S3.TIF]

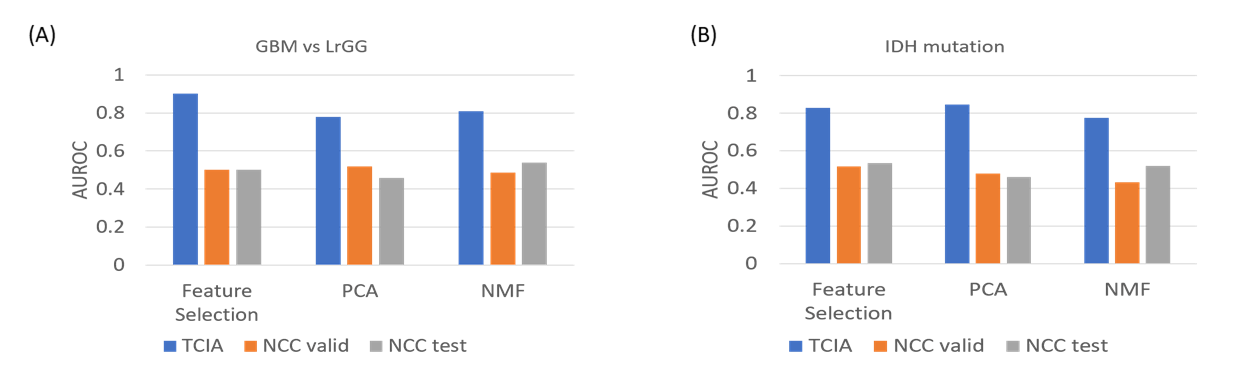

Supplement: Supplementary file 1 [file cancers-13-03611-s001.zip › Supplementary_Figure_S4.tif]

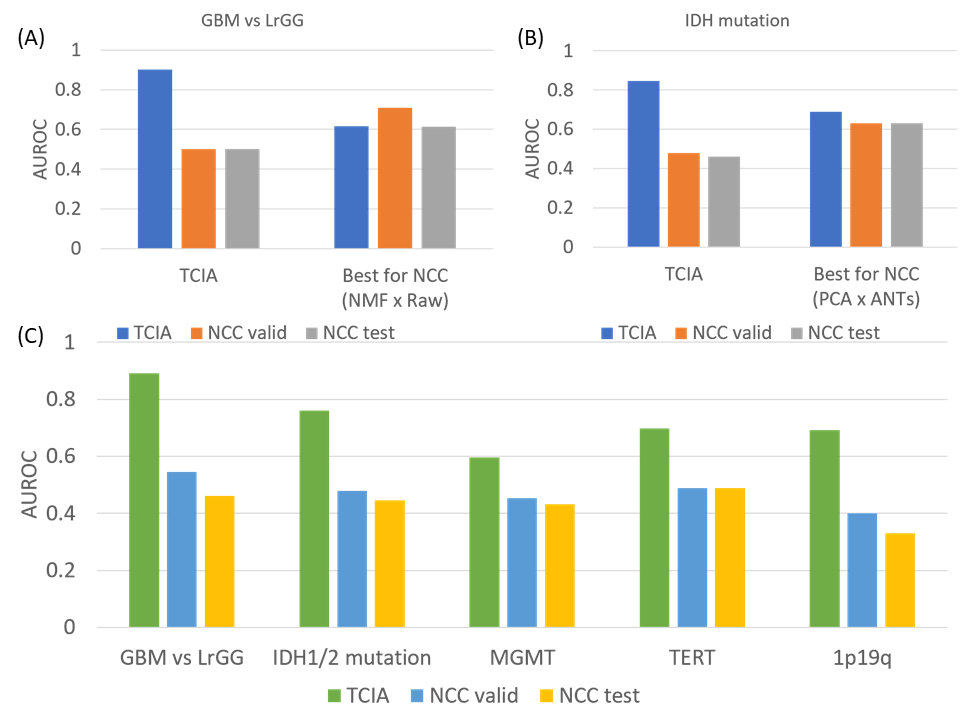

Supplement: Supplementary file 1 [file cancers-13-03611-s001.zip › Supplementary_Figure_S5.tif]

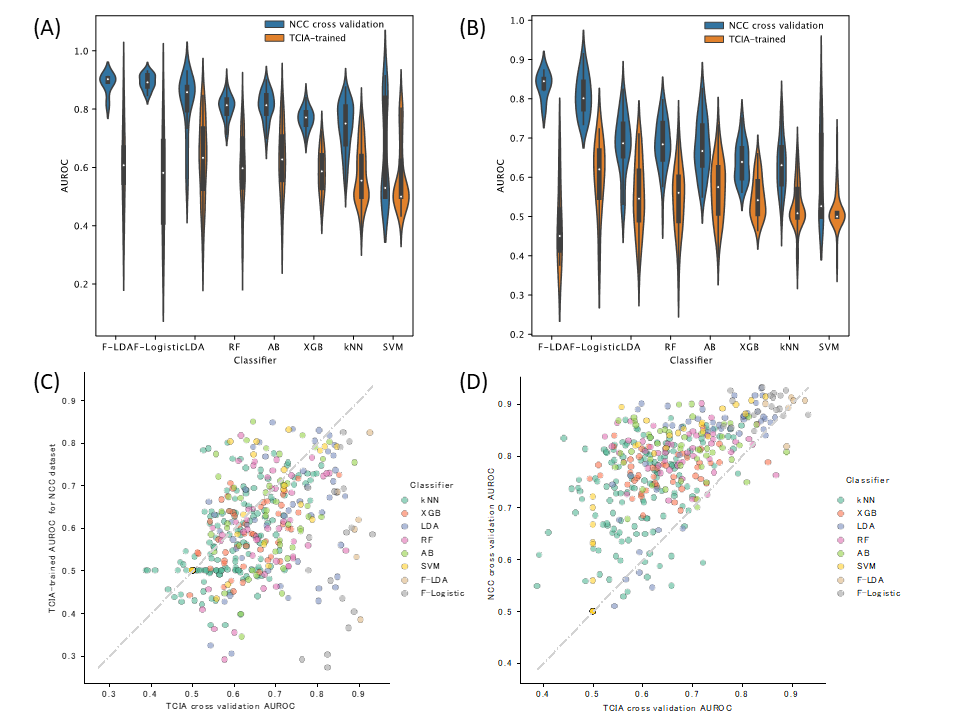

Supplement: Supplementary file 1 [file cancers-13-03611-s001.zip › Supplementary_Figure_S7.tif]
